# Endometrial cancer tissue features clusterization by kurtosis MRI

**DOI:** 10.1002/mp.17718

**Published:** 2025-02-28

**Authors:** Alessandra Maiuro, Francesca Di Stadio, Marco Palombo, Andrea Ciardiello, Serena Satta, Angelina Pernazza, Martina Leopizzi, Carlo Della Rocca, Carlo Catalano, Lucia Manganaro, Silvia Capuani

**Affiliations:** ^1^ Department of Physics Sapienza University of Rome Rome Italy; ^2^ Physics Department Rome CNR ISC Roma Sapienza Rome Italy; ^3^ Cardiff University Brain Research Imaging Centre (CUBRIC) School of Psychology Cardiff University Cardiff UK; ^4^ School of Computer Science and Informatics Cardiff University Cardiff UK; ^5^ Istituto Superiore di Sanità Rome Italy; ^6^ Department of Radiological, Oncological and Pathological Sciences Umberto I Hospital Sapienza University of Rome Rome Italy; ^7^ Fondazione Santa Lucia IRCCS Rome Italy

**Keywords:** diffusion kurtosis imaging, endometrial cancer, tissues clustering

## Abstract

**Background:**

Endometrial cancer (EC) is one of the most common gynecological malignancies and the second most common gynecological malignancy cause of death in women. Heterogeneous tissues with different grades of complexity and different diffusion properties characterize the EC. Several diffusion magnetic resonance imaging (DMRI) protocols have been used to perform a non‐invasive and global evaluation of EC for diagnostic and prognostic purposes. However, the association of a single value for the diffusion coefficient to an EC tissue could be a severe limit for developing a DMRI virtual histology protocol.

**Purpose:**

This study evaluates the potential of diffusion kurtosis imaging (DKI) and tissue multiple diffusion clusterization in detecting the specific features of healthy/cancer tissue that can be useful in EC diagnosis and prognosis.

**Methods:**

Thirty‐eight subjects were analyzed: 18 with a final diagnosis of EC and 20 healthy, asymptomatic, with no history of endometrial pathology and uterine tumor pathology. Diffusion‐weighted Spin‐Echo Echo‐Planar Imaging (DW‐EPI) with TR/TE = 2000 ms/77 ms was used at 3T using six different *b*‐values: (500, 800, 1000, 1500, 2000, and 2500)s/mm2 along three gradient directions (*x*, *y*, *z*). The decay of the signal in each voxel was used to obtain clusters of different diffusion compartments reflecting tissue heterogeneity. Moreover, using the Kurtosis representation, the parametric maps of the apparent kurtosis (K) and diffusivity (D) coefficients were obtained. The statistical analysis of the differences in the mean value of the parameters obtained in the selected regions of interest (ROIs) in tumor area (T) peritumor area (PT) and healthy tissue was carried out using a Kruskal–Wallis Test. A *p*‐value < 0.05 indicated a statistically significant difference. To validate DKI and multiple diffusion clusterization in the detection of EC and healthy tissue, DMRI results were compared with EC histology. A ROC curve analysis was performed to evaluate the performance of the clustering feature in differentiating healthy and tumoral tissues.

**Results:**

K discriminates the peritumor area (PT) of the tumor from the healthy tissues (*p* < 0.05) and the area inside the EC (cancerous tissue, *p* < 0.05). This result is validated and explained by the diffusion clustering, which shows a great variability in K for pathological compared to healthy subjects. Moreover, the standard deviation of K in the cluster defined by the highest K/D ratio differentiates T and H ROIs.

**Conclusions:**

K as well as diffusion clusterization are sensitive to the different microstructural organizations in EC and healthy tissue, promoting themself as a potential tool for the diagnosis and prognosis of EC.

## 1 INTRODUCTION

Endometrial cancer (EC) is the second most common gynecological malignancy in developed countries^1^ and it is the sixth most common cancer worldwide for women.^2^ In Western Europe, it is the seventh cause of cancer‐related deaths in the female gender. Diagnosis and preoperative prognosis are essential to perform better surgical procedures and implement a suitable therapy as quickly as possible. Current clinical protocols for the diagnosis of endometrial carcinoma are based on invasive gynecological techniques, such as biopsy and curettage.

Traditionally, this type of carcinoma has been classified as Type I or Type II based on clinical, endocrine, and epidemiological observations (Bokhman subdivision). Nowadays, a new classification based on molecular stratification is used.^3^, ^4^ However, diagnostic uncertainty can occur in the histological examination of the biopsied tissue due to the operator's experience and the heterogeneity of the carcinoma. Indeed, the operator takes only a portion of the tumor mass and, therefore, the examination could highlight only local and non‐general characteristics. Consequently, inaccurate knowledge of the pathological degree of the tumor can negatively influence the outcome of the disease.

Magnetic Resonance Imaging (MRI) may be able to characterize the tumor lesion in all its extension, in a non‐invasive and radiation‐free modality. In this context, diffusion magnetic resonance imaging (DMRI) showed great potential for the diagnosis and prognosis of EC.^5^, ^6^, ^7^ Some works highlighted that the apparent diffusion coefficient (ADC) values can distinguish EC from healthy endometrial tissues or benign endometrial lesions.^8^, ^9^ Other authors showed that ADC values can assess the degree of cancerous cells’ differentiation.^10^ In the last years, DMRI investigations based on diffusion‐weighted measurements at different diffusion weights (*b*‐values) were developed to increase the sensitivity and specificity of diffusion investigations. Among these, Diffusion Kurtosis Imaging (DKI)^11^ that allows the quantification of kurtosis parameter (K) and diffusion parameter (D) has shown interesting results in different investigations related to neurological pathologies such as the evaluation of brain gliomas^12^, ^13^, ^14^, ^15^ and the evaluation of meningiomas cell proliferation.^16^ Recently the DKI has also been used for the study of the body. For example, K quantified in the prostate provides a better identification and classification of prostate cancer^17^, ^18^ compared to other MRI investigations. However, as DMRI is an indirect measure of the medium microstructure and relies on parameters’ inference from representations or models based on specific assumptions, it is necessary to validate the information obtained from these methods through histological investigations.^19^

Heterogeneous tissues with different grades of complexity and different diffusivity properties characterize the EC. The association of a single K or D value to an EC could be a severe limit for developing a DMRI virtual histology protocol. Therefore, in this study, to extract and highlight multiple diffusion tissue characteristics that may lead to more detailed information on the histological nature of cancer itself, a differentiation based on the k‐means clustering was performed on the data obtained by the kurtosis model.

Regarding the characteristics of the lesion to be evaluated to obtain useful information for diagnosis and prognosis, recent studies on soft‐tissue sarcoma have found the peritumoral tissues’ characteristics to be associated with the tumor's malignancy.^20^, ^21^, ^22^ Despite the differences in tissues between sarcomas and adenocarcinomas, Deng et al.^23^ found significant differences between deep and superficial myometrial invasion in peritumoral regions of EC, confirming the importance of further investigation on peritumoral tissues.

The present study aimed to evaluate the potential of DKI in detecting the specific features of healthy and EC tissues, to be useful in EC diagnosis and prognosis. For this purpose, DKI results were validated and explained using diffusion clusterization and histological outcomes. Furthermore, diffusion parameters were tested in the tumor area (T), in the area of the healthy endometrium (H), and in the area immediately outside the tumor endometrium (peritumor, PT).

## 2 MATERIALS AND METHODS

**FIGURE 1:**
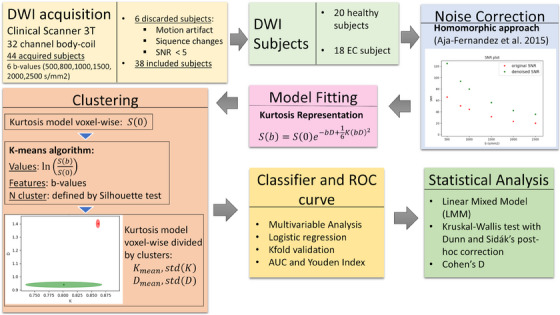
The pipeline of the analysis performed in this work.

The pipeline of this study is displayed in Figure 1.

### 2.1 Subjects

The prospective observational study was approved by the Ethical Committee of the University Hospital Policlinico Umberto I (Rome, Italy). All patients signed the informed consent for the experimental protocol. Eighteen patients with a final diagnosis of EC were investigated (age range: 51–87 years, mean ± standard deviation (SD): 72 ± 10 years, BMI range: 23–43Kg/m^2^, average ± SD: 30 ± 5Kg/m^2^). In particular, three subjects were classified as Endometrioid with grade G1, six as G2, five as G3, and four as serous papillary (grade G3 serous). In addition, 20 subjects were enrolled as a control group with a H (age range: 47–79 years, mean ± SD: 59 ± 7 years, BMI range: 21–31Kg/m^2^, mean ± SD: 25 ± 3Kg/m^2^). Both EC subjects and healthy volunteers were in a menopausal state (see Table S1 in Supplemental Material for subjects’ specifications). EC patients with the presence of further contemporary or past neoplastic pathologies or undergoing chemotherapy or radiotherapy treatment were discarded in this work.

Histology of patients’ tissue extracted after surgical treatment consisting of bilateral hysterosalpingo‐oophorectomy was obtained as described by Satta et al.^9^

### 2.2 MRI acquisition protocol

All patients and healthy volunteers underwent an MRI performed using a 3.0 T clinical scanner (GE Discovery MR 750 3.0 T—GE Healthcare, Milwaukee, WI, USA). Images were acquired using a Diffusion‐weighted Spin‐Echo Echo‐Planar Imaging (DW‐EPI) with TR/TE = 2000 ms/77  ms; bandwidth = 1953  Hz; matrix size = 256 × 256, FOV 300 × 300mm^2^. The number of slices varied from 9 to 11 (depending on endometrium extension) for all the healthy subjects and the EC patients, except five healthy subjects and five EC patients, for which the number of slices varied from 11 to 34. Since the repetition time TR also depends on the number of slices, the images of these 5 healthy subjects and 5 EC patients had different TR: between 4s and 6s. The in‐plane resolution was 1.17 × 1.17mm^2^, and STK = 5 mm. The diffusion encoding gradients were applied along three no‐coplanar directions using six different *b*‐values: (500, 800, 1000, 1500, 2000, and 2500) $s/mm^{2}$ and averaged over the three directions. The number of averaged signals (NS) for each *b* value was NS = 2. Acquisitions were obtained using a body coil with 32 channels positioned on the lower abdomen and a SENSE accelerator algorithm. The second‐order spherical harmonic shim correction was used.

### 2.3 Regions of interest

**FIGURE 2:**
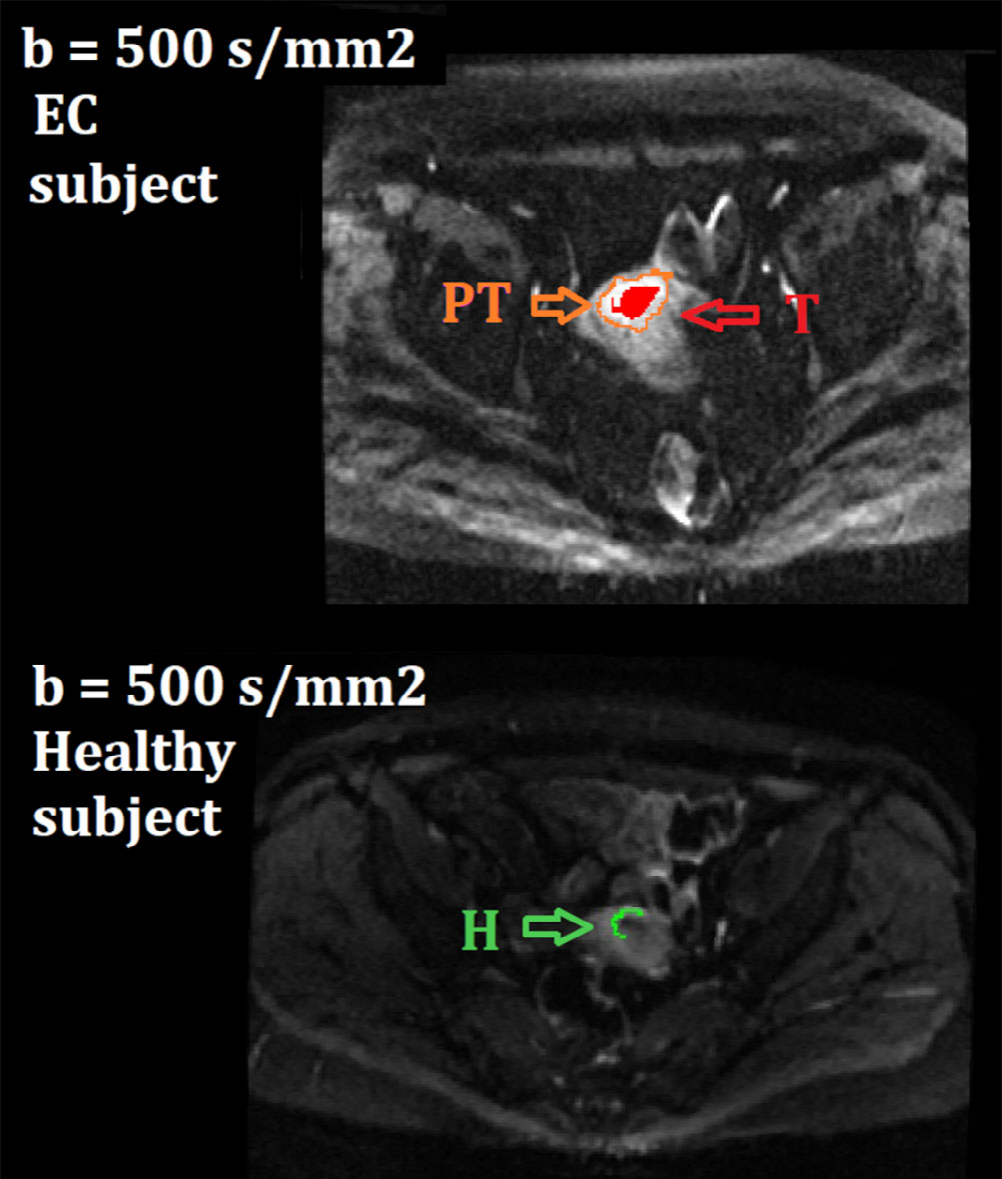
ROI segmentation of tumoral (red), peritumoral (orange) and healthy tissue (green). ROI, region of interest.

In this study, three different regions of interest (ROIs) were considered as shown in Figure 2: (1) ROI of the T, (2) ROI of the peritumor area (PT), and (3) ROI of the H only on healthy subjects. The ROI segmentation was performed jointly by two specialist radiologists with 4‐ and 20‐years of experience, respectively. Moreover, to have further confirmation of the correctness of the positioning, the ROIs were identified using both diffusion and T2‐weighted images.

Finally, an ROI on the bladder was segmented to evaluate the background level of noise obtained in the DW acquired at b = 2500s/mm^2^.

### 2.4 Data processing

It is well known that the kurtosis parameters are influenced by the noise.^24^ Therefore, the Signal‐to‐Noise‐Ratio (SNR) was evaluated for each patient, considering as signal the median of S(b) in all the ROIs (H, PT, and T) and keeping as noise the SD of the signals at b = 2500 $s/mm^{2}$ taken on the bladder ROI. Indeed, the bladder contains urine, which can be considered like free water with faster diffusion compared to the surrounding tissues’ diffusion; thus, it has no signal for high *b*‐values. The choice of using the bladder's signal instead of the background comes because images have no available background zones.

Finally, a denoiser tool based on a homomorphic approach for the spatially variant noise estimation developed by Aja‐Fernandez et al.^25^ was applied to the images to reduce/eliminate the K dependence on the noise. The original adopted toolbox is available on MATLAB.^26^

### 2.5 Conventional kurtosis representation fitting

The kurtosis representation was fitted to the averaged voxel signal values within each ROI using the following function to describe the signal decay^11^, ^27^:

(1) $$S\;\left(b\right)=S\left(0\right)e^{\left(-bD_{app}+\frac{1}{6}b^{2}D_{app}^{2}K_{app}\right)}$$

where S (b) is the signal as a function of *b*‐values $b={\left(\gamma g\delta \right)}^{2}\;\left(\Delta -\frac{\delta }{3}\right)$, S (0) is the signal at b = 0 $s/mm^{2}$, $D_{app}$ (in $mm^{2}/s$) represents the ADC corrected for non‐Gaussian bias and $K_{app}$ (dimensionless) represents the apparent kurtosis coefficient, that is, the deviation from the Gaussian behavior.^11^, ^27^ For simplicity, in this paper, we will refer to $D_{app}$ and $K_{app}$ as D and K, respectively.

The fitting was performed adopting a homemade Python script using the nonlinear least square method (“SciPy” library, scipy.org) and the following boundary constraints: K ∈ [‐0.7,3.7] and D ∈ [0.01,3]e‐3 mm^2^/s.

The parametric maps of K and D of the Kurtosis model were obtained using a voxel‐wise fitting procedure adopting the same homemade Python script.

### 2.6 Identification of multiple components with a k‐means clustering

Multiple diffusion tissue characteristics were obtained by k‐means clustering of the data using Python's library “sklearn” (scikit‐learn.org).

To individualize the signal's clusters, the kurtosis representation was also fitted voxel‐wise on each ROI to obtain an estimation of the signal at b = 0, $S_{e}\left(0\right)$, then the voxels’ signals S(b) were normalized by the interpolated $S_{e}\left(0\right)$ to uniform the signals before clustering. The k‐means was performed on the logarithmic normalized signals $\ln(\frac{S(b)}{S_{e}\left(0\right)})$ with specified number of clusters based on the silhouette test.^28^ Since the mean number of defined clusters was 2, finding that only 2 pathological and 3 healthy subjects showed 3 clusters (Figure S1 in Supplemental Material), the clustering algorithm was applied fixing the number of clusters to two to maintain the results as general as possible.

Voxel‐wise fitting of the kurtosis representation was performed on the original signals S(b) in each cluster so that the mean and SD of the diffusion and the kurtosis parameters could be evaluated for each cluster for both pathological and healthy subjects’ groups. The labels of the clusters were named “Cluster 1″ for the cluster with the minimum ratio K/D and ”Cluster 2″ for the cluster with the maximum ratio K/D.

The voxel‐wise fitting was also performed considering the entire ROIs as a single cluster to evaluate the advantage of clustering with two clusters.

### 2.7 Multivariable analysis and ROC curve

We considered four different models: (1) the conventional Kurtosis representation, where features are D and K on each ROI; (2) the clustering model where the following twelve features were analyzed: $D_{1},\;K_{1},stdD_{1},\;stdK_{1},\;\;KDratio_{1}=K_{1}\;/D_{1}$ for the Cluster 1 (where $stdD_{1}$ and $stdK_{1}$ are the SD of the diffusion coefficients and the kurtosis of the cluster 1), $D_{2},\;K_{2},stdD_{2},\;stdK_{2},\;\;KDratio_{2}=K_{2}\;/D_{2}$ for the Cluster 2, $D_{ratio}=D_{1}\;/D_{2}$ and $K_{ratio}=K_{1}\;/K_{2}$; 3) the clustering model assuming only one cluster on the entire ROI with the following five features: D,K,stdD,stdK,KDratio=K/D; 4) the clustering model considering one cluster at the time with the five features defined before. The datasets were divided into five folds and a univariate feature selection was performed individually for each fold testing for their separation power using a Mann–Whitney test with a statistical significance set at p≤0.05/n with n the number of the model's features, according to Bonferroni correction. Once sorted by increasing p‐value, the best‐uncorrelated features (Pearson correlation ρ<0.8) were selected for the multivariable analysis. Features were standardized using the *z*‐score, and then logistic regression with Lasso regularization was performed.^29^ AUC score was evaluated over the folds and the confidence interval was obtained by performing an a‐posteriori bootstrapping with 1000 bootstraps. We reported the performances of the logistic regression as accuracy, precision, recall, and f1 score computed at the Youden Index. Results are reported as median with a confidence interval between 5% and 95% over the folds.

### 2.8 Statistical analysis

Since in principle, patients differentiate in tissue type but also in SNR level, age, and TR, the following linear mixed model (LMM) was performed on the kurtosis model's parameters K and D, using the MATLAB function *fitmle* (Matlab 2021a), to evaluate the actual dependency of K and D on all these confounders:

y∼βX+zu+ε

where y is the parameter's vector, X is a matrix of the fixed effect (tissue's type, SNR level, age and TR), β is the fixed effect coefficient, zu is the random effect contribute including the tissues’ type corrected by the tumor grade, the SNR corrected by the tissues (since there is a difference in the T2 relaxation time^30^), and the correction of the intercept for ages. Finally, ε is the random error. The coefficients obtained by the LMM have *p*‐values assessing if they are significantly different from zero. These *p*‐values were corrected following the Benjamini and Hochberg method.^31^

The Kruskal–Wallis test (Non‐parametric Analysis of variance) with Dunn and Sidak's correction (Matlab 2021a) and Cohen's d effective size were performed to evaluate the significant differences between the average parameters obtained in the three areas (T, PT, H). A *p*‐value < 0.05 was selected for the statistical significance.

## 3 RESULTS

In general, the denoised images gained more than 70% of SNR for each *b*‐value to the original DWIs. The minimum SNR at *b* value 2500 $s/mm^{2}$ was about seven in denoised images.

The LMM applied on denoised images reflects no dependencies of the kurtosis parameter K on SNR, TR, and patients’ age, whereas it showed a dependence on the tissue type with a corrected *p*‐value < 0.05 (Table 1). On the other hand, D seems to depend on the intercept with a high coefficient and the SNR with a small but significant coefficient (Table 1).

**TABLE 1:** Linear mixed model output for K and D parameters obtained by denoised images. *p*‐value BH is the corrected *p*‐value with the Benjamini and Hochberg method.

Parameter	Name	Estimate	*p*‐value	*p*‐value BH
K	Intercept	0.8	0.1	0.2
	SNR	−0.003	0.2	0.3
	Tissue	0.9	0.00003	0.0001
	TR	−0.08	0.1	0.2
	Age	−0.0004	1	1
D	Intercept	0.7	0.003	0.008
	SNR	0.002	0.0004	0.002
	Tissue	−0.07	0.1	0.2
	TR	0.02	0.5	0.7
	Age	0.0004	0.9	0.9

Abbreviations: SNR = signal to noise ratio, TR = repetition time.

### 3.1 Conventional kurtosis representation

**FIGURE 3:**
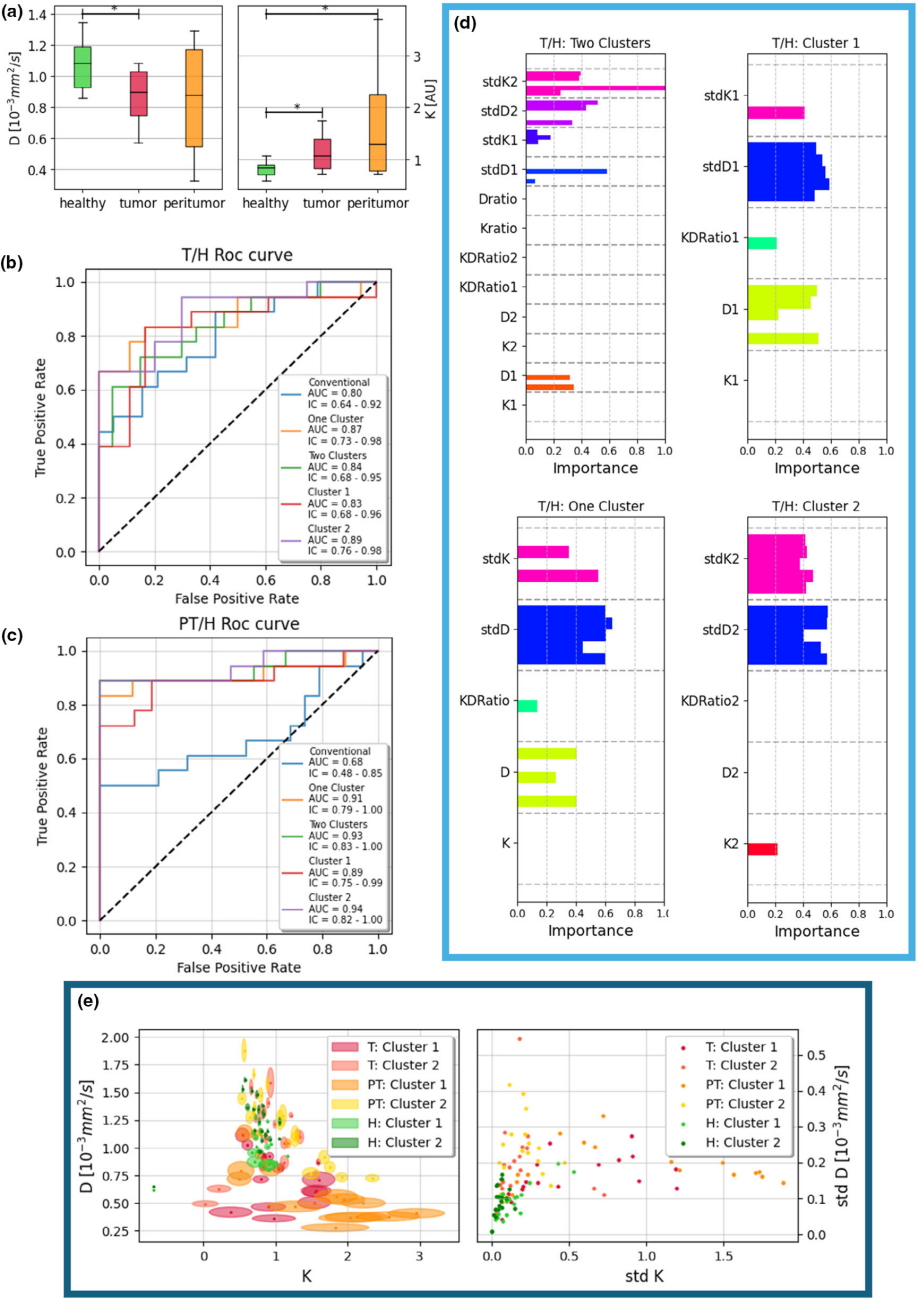
(a) boxplot of diffusion coefficient D (*10^−3^ mm^2^/s) and kurtosis parameter K evaluated in the ROIs of healthy, tumoral, and peritumoral tissue. *p < 0.05. (b) ROC curve for the healthy‐tumoral separation. The conventional model is the conventional kurtosis representation application; One cluster is the model where the ROI is treated as a single cluster; Two Clusters: the features belonging to the clusters are considered; Cluster 1: only Cluster 1 is considered; Cluster 2: only Cluster 2 is considered. (c) ROC curve for the healthy‐peritumoral separation. (d) bar‐plot of the normalized coefficients for the logistic regressor indicating the importance of each feature in the multivariable analysis in T/H comparisons. (e) On the left: clusters’ map obtained for healthy tissues (H), tumor area (T), and peritumor area (PT); on the right: scatter plot of the kurtosis model's parameters K and D SD found in healthy, tumoral and peritumoral tissues.ROI, regions of interest; SD, standard deviation.

The Kurtosis coefficient K obtained in both the PT and the T zones is significantly higher than that obtained in the H zone (*p*‐value = 0.01 and *p*‐value = 0.05, respectively, see Figure 3a and Table S2). The diffusion coefficient D calculated in the T zone is significantly lower than in the H area (*p*‐value = 0.03, Figure 3a and Table S2). In Table S3 of Supplemental Material is available the Cohen's d values for each ROI's difference reflecting the Kruskal–Wallis results with the high effect size for the Kurtosis K differences between the PT and T zones compared to the H area and the diffusion coefficient D between the tumoral tissues T and the normal tissues H.

### 3.2 K‐means clustering

**FIGURE 4:**
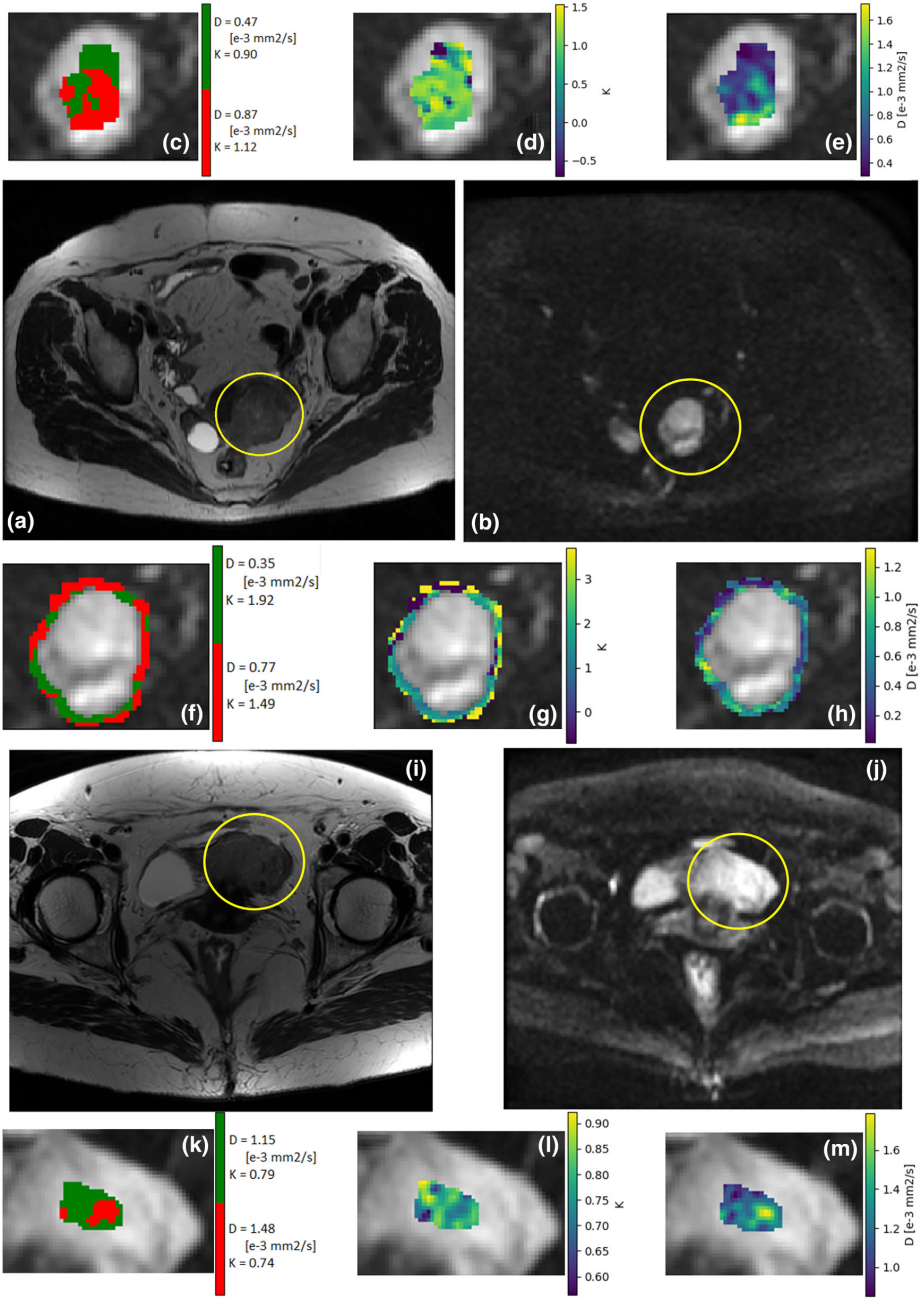
(a) 73‐year‐old patient with grade 1 (G1) endometrioid adenocarcinoma. a) the T_2_ image shows a slightly higher signal intensity in cancer. (b) a diffusion‐weighted image, DWI (b value = 500 $s/mm^{2}$); (c) DWI shows the clusters’ labels on the tumoral ROI. (d) the parametric map of the diffusion coefficient D shows a low value of D within the tumor. (e) parametric map of the kurtosis coefficient K shows a high value of K in the T ROI; (f) clusters’ labels on the peritumoral ROI; (g) D map on the PT ROI; (h) K map on the PT ROI; (i) T_2_ image of a 65‐year‐old healthy subject; (j) DWI (*b*‐value = 500 $s/mm^{2}$); (k) clusters’ labels on the healthy ROI; (l) D map on the H ROI; (m) K map on the H ROI. ROI, region of interest

The k‐means clustering algorithm individualized two main diffusive compartments for both healthy and pathological subjects, as shown in the parametric maps displayed in Figure 4 (in Figure S3 of Supplemental Material, the histologies of the tumoral subject are available). Figure 3e shows the clustering map obtained by the k‐means analysis. In the left panel of Figure 3e, each point represents the mean values of the parameters couple (K; D) in each cluster, whereas the axes of the surrounding ellipse represent half of their SD. Healthy subjects (green marks) are concentrated in the center of the plot, with barely visible ellipses meaning that the couple (K; D) has no significant variability inside the healthy tissues. The T and PT zones show instead greater SD and various values for the couple (K; D). In particular, most of the variability was found for the K parameter in the peritumoral ROIs (orange and yellow dots in Figure 3e). In the right panel of Figure 3e, the scatter plot of the SDs summarizes these results showing all the healthy green dots on the bottom left of the plot, whereas the SDs of the pathological tissues’ compartments are spread along the K axis. In general, the approximate ratio of voxels belonging to Cluster 1 was 50% (i.e., equal to the ratio in Cluster 2) compared to the entire ROI.

The ROC curve in Figure 3b shows an AUC (95% IC) = 0.84 (0.68–0.95) for the identification of tumoral zones compared to the healthy tissues using the Two Clusters model with fifteen features, while ROC curves of the model considering the Single Cluster 2 showed the best AUC score = 0.89 (0.76–0.98). In Figure 3c, the ROC curve of the four analyzed models for the identification of the peritumoral ROI shows the best AUC = 0.94 (0.82–1.00) for the Single Cluster 2. The bar‐plots in Figure 3d show the normalized weight adopted during the linear regression of the classifier for each fold and feature for the models with more than two features, indicating stdK2 and stdD2 as the features that most diversify tumoral and healthy tissues (in Supplemental Material, the bar‐plots for the PT ROIs are displayed in Figure S2). In Table 2, the median values of the classification parameters averaged over the 5‐fold validation are reported, while the results for each fold are displayed in Table S4 of Supplemental Material for the best model Single Cluster 2.

**TABLE 2:** Parameters obtained by the classification metrics averaged over a five‐fold validation for the best model of single cluster 2 at Youden index.

	Median	5% CI	95% CI
T/H			
Accuracy	0.86	0.77	0.98
Precision	1	0.76	1
Recall	1	0.53	1
F1 score	0.86	0.69	0.98
AUC	0.89	0.76	0.98
PT/H			
Accuracy	1	0.90	1
Precision	1	0.84	1
Recall	1	1	1
F1 score	1	0.91	1
AUC	0.94	0.82	1

Abbreviation: CI = confidence interval.

## 4 DISCUSSIONS

In this paper, we achieved some new important information on EC heterogeneity and histopathology by clustering the signals inside endometrial tissue ROIs. We found that the parametric maps of the kurtosis coefficient K and the diffusion coefficient D show comparable values distribution with those obtained by the clustered signals also showing a great variability of values in pathological tissues compared to healthy ROIs. In general, greater K values are associated with increased tissue complexity^11^ but also with increased magnetic susceptibility difference at the interface of different tissues.^32^ A higher value of K in the tumor area (T) compared to the average value obtained in the H can be explained as cancer cells create a more chaotic and complex environment than the typical structure of a healthy tissue. Instead, the D maps show lower values of diffusion coefficient D in the T. This is in agreement with the slower water diffusion within the cancerous tissue compared to the average diffusion in healthy tissue which is consistent with the cell density increase in endometrial carcinoma.^10^, ^33^

From a histological point of view, the tumor tissue is characterized by a huge and uncontrolled growth of cells, which creates a denser and more disordered environment with cells of different sizes.^34^ The clusterization highlighted these same features, pinpointing a greater variability in K and D parameters in pathological than in healthy tissues. Indeed, the SD's scatter plots show a clear variability in K for pathological tissues, especially in the PT zone where different factors such as the tumor infiltration and the magnetic susceptibility difference^32^ may contribute to the voxels’ intensities. Moreover, the best classifier showed positive coefficients in the features’ selection for stdD2 and stdK2. These results suggest Cluster 2 (defined as the one with the highest K/D ratio, hence higher complexity and hypercellularity) to be the one responsible for the classification as tumoral tissues.

Although results are in general agreement with the literature,^9^, ^35^, ^36^ the paper by Yue et al.^36^ reports K and D values, obtained both in zone T and in zone H, slightly different from the results obtained in this paper. In particular, Yue et al. and Yamada et al.^35^ suggest a significant positive correlation between K values and tumor grading that we have not investigated in this paper given the small cohort of patients for each grade. Moreover, the scanner's field strength used by Yamada et al.^35^ was 1.5T, whereas our patients were acquired using a 3T scanner. However, in our opinion, the factors that mostly lead to different results between the present work and the previous ones are related to the great variability and typology of EC, how the histologies were obtained to establish the classification of the tumor, the fact that we evaluated the diffusion parameter inside the tumor without the boundaries (T ROI) and only in the boundaries (PT ROI), and most important, the size of our subjects’ cohort which was too small. Moreover, all these differences revealed that reliable threshold values for clinical diagnosis are still not defined and all these inconsistencies may be overcome by changing the methodology. Indeed, our main aim was not to show the effectiveness of a diagnostic protocol but to highlight that diffusion‐based clustering improves the quantification of the heterogeneity observed in tissue histology, and together they explain the kurtosis and Diffusion values in EC and healthy tissues.

We found statistically significant differences between the Kurtosis parameters obtained in the peritumoral area (PT). The clustering analysis also underlined the huge variances in these tissues for PT zones, especially for the kurtosis parameter K, supporting our results. Moreover, Palombo et al.^32^ showed that the parameter K is positively correlated to the magnetic susceptibility difference at the interphase between different tissues. According to this observation, we obtained higher values of K in PT ROIs, that is, in those ROIs at the boundaries between cancer and healthy tissue.

As confirmed by the clustering analysis, the tumor area (T) is highly inhomogeneous due to the presence of some areas of necrosis. At the histological level (micron scale) the necrotic cells and the neoplastic cells are separated and they constitute zones of cellular order and disorder, respectively. However, within a voxel (millimeter scale) the contributions of these two different types of cells can be averaged. Therefore, it is reasonable that a significant difference in diffusion with the grade of the tumor has not been found in the tumor area (T).

The main limitation of this study is related to the small cohort of subjects (38 subjects) and a wide variety of endometrial tumor types whose ROIs were manually placed. Regarding the ROIs, they were selected jointly by two experts in EC diagnostics. Inter‐operator variability was therefore not assessed. Moreover, images averaged over the three‐diffusion directions may lead to systematic errors in the K parameter estimation,^37^ especially in tissues with low or moderate diffusion anisotropy. Despite these limitations, this study aimed to highlight the clustering method that seems to discriminate healthy and tumoral tissues with high specificity and accuracy, providing a promising tool for EC diagnosis. Future research will include a larger cohort to establish threshold values for diffusion, kurtosis, and cluster counts to support the diagnosis and prognosis of EC. Finally, the clustering method aligns with the principles of precision medicine, a rapidly growing area of interest.

## 5 CONCLUSION

The present study showed the potential of DKI and diffusion clusterization analysis in providing tissue features for a non‐invasive diagnosis of endometrial carcinoma. The kurtosis coefficient K, which describes the heterogeneity and complexity of the tumor tissue formed by the uncontrolled and chaotic proliferation of cancer cells, further discriminates the area immediately outside the peritumor area (PT) from the area of H. These results are validated by the clustering process which underlines the great variability in K of the pathological tissues and, especially their edges. In particular, the K parameter and its SD provide important information on the contour and possible tumor expansion. Indeed, tumoral tissues are differentiated from healthy subjects by the SD of K belonging to the cluster with the highest K/D ratio. Therefore, the K parameter corroborated by diffusion clusterization could be a useful tool for an early diagnosis of EC and may be employed to optimize the prognosis providing an identification of the nature of the specific EC.

## CONFLICT OF INTEREST STATEMENT

The authors declare no conflicts of interest.

## Supporting information



Supporting Information.

## Data Availability

Data are not available.
