# Supplementary material for: Endometrial cancer tissue features clusterization by kurtosis MRI
Source: Med Phys. 2025 Feb 28;52(5):2898–908. doi: 10.1002/mp.17718 (PMC12059522; doi:10.1002/mp.17718)
Supplement: Supplementary file 1 — Supporting Information. [file MP-52-2898-s001.docx]

**Supporting Material**

**Supplementary FIGURES**


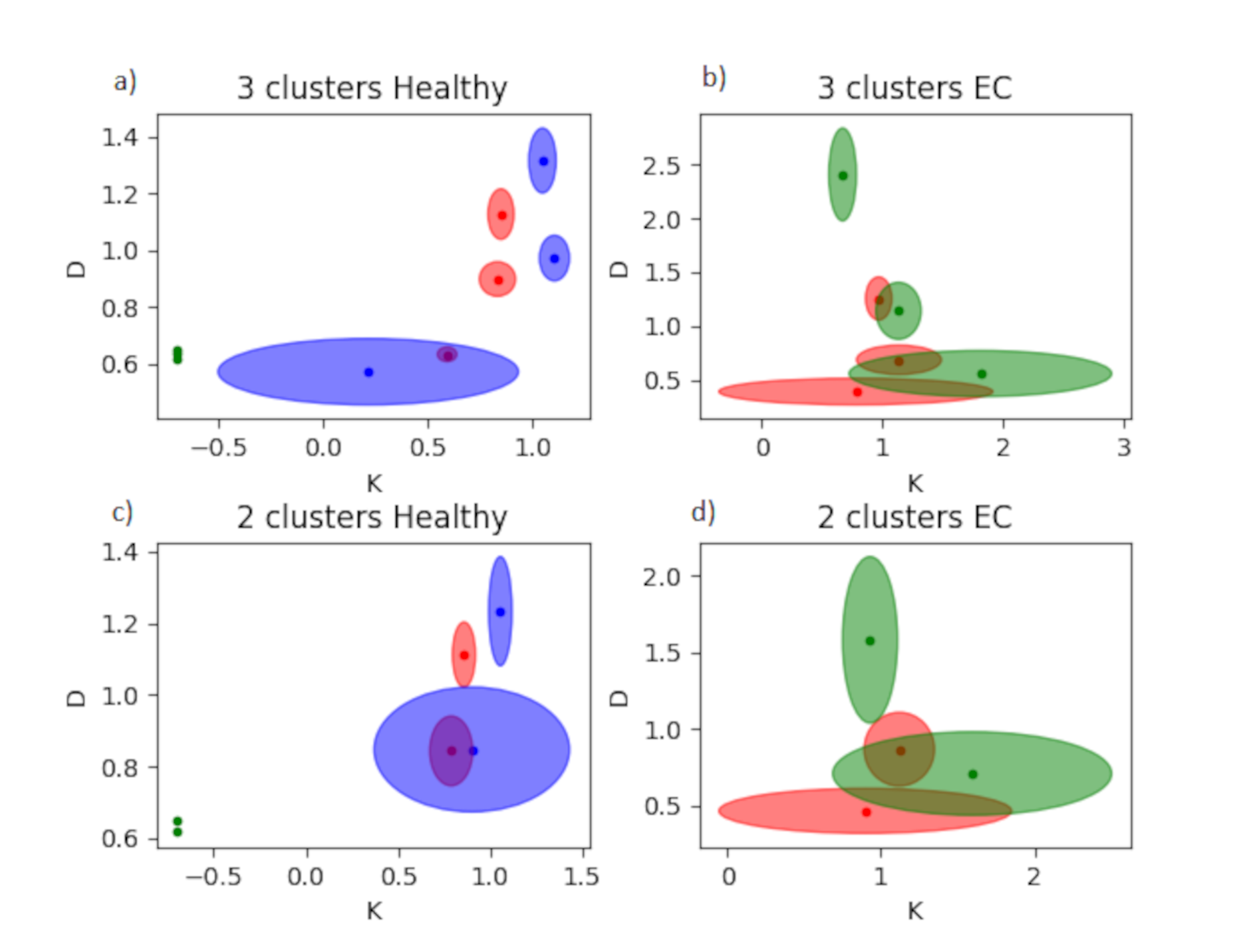


**Figure S1** a) plot of three healthy subjects (red, green, and blue) clustered in three groups; b) plot of two pathological subjects (red, and green) clustered in three groups; c) plot of the same three healthy subjects of a) clustered in two groups; d) plot of the same three EC subjects of b) clustered in two groups.


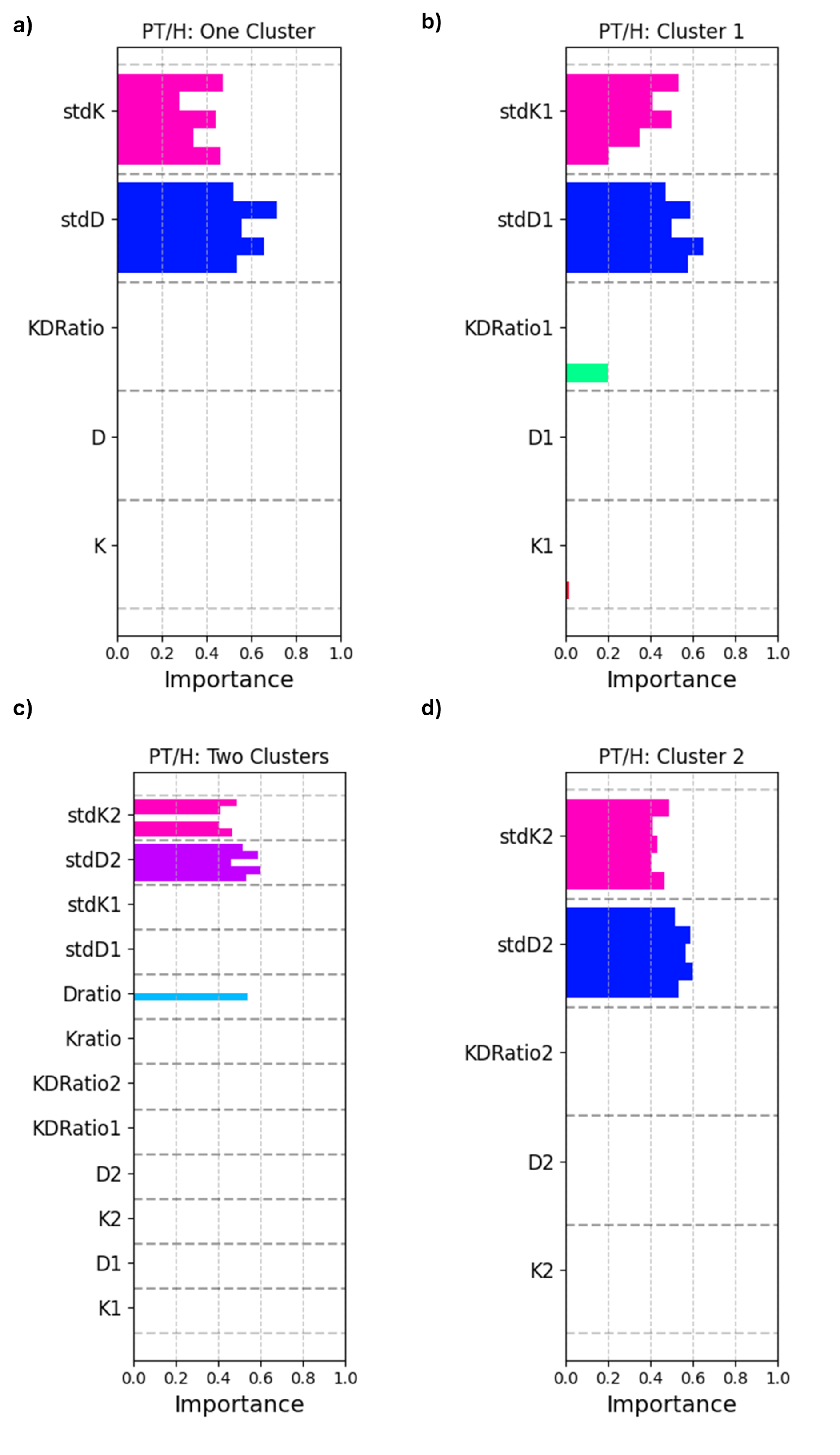


**Figure S2** bar-plot of feature importance for classification between peritumoral ROI and healthy tissue. Standard deviation of D and K, in particular of D and K belonging to Cluster 2, are the most important features for the classification.


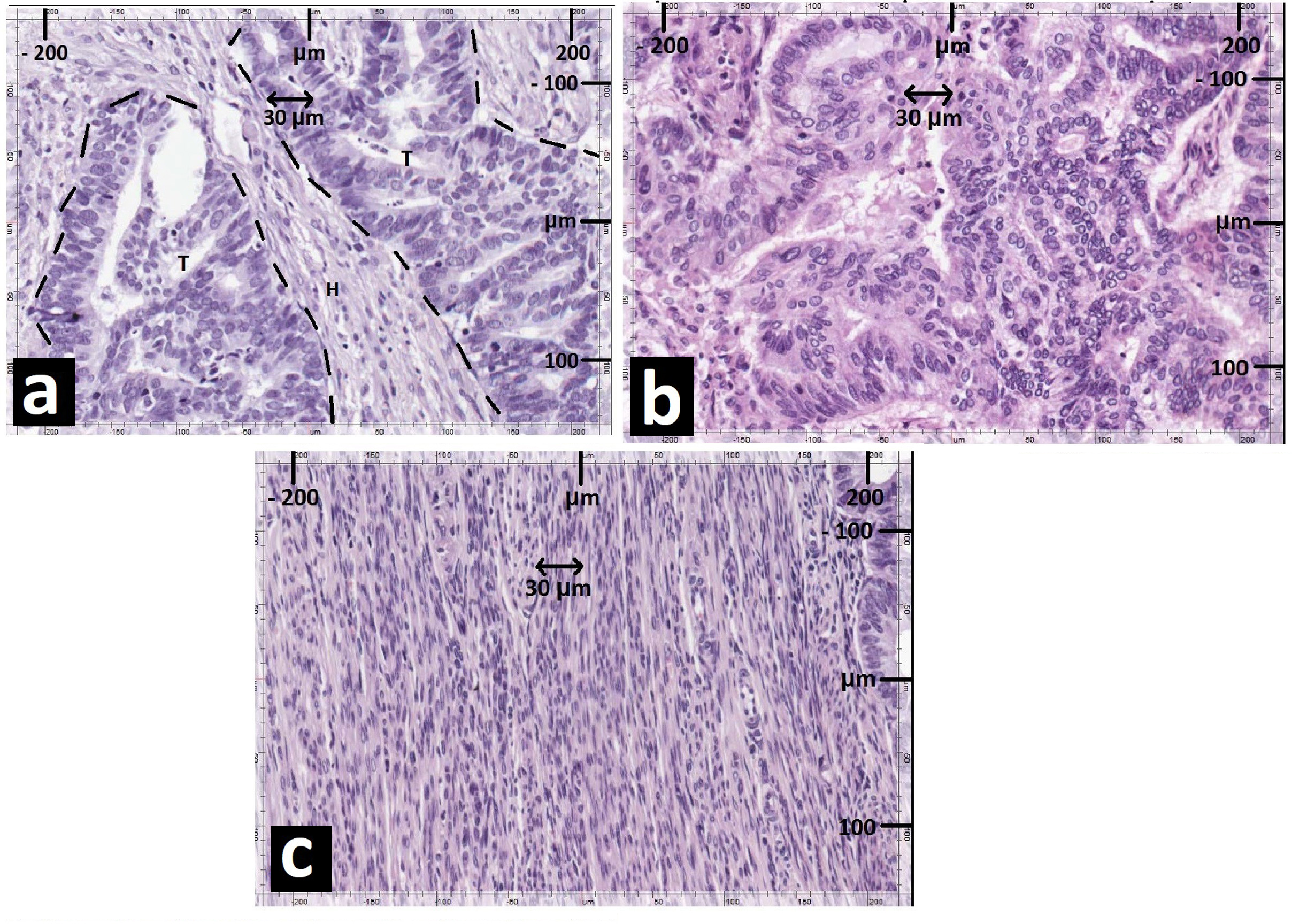


**Figure S3** The figure displays the histology of the G1 tumor analyzed in Fig. 4 of the paper showing that the architectural patterns are cribriform, moderate is the desmoplastic reaction. At the same time, the area interested by the necrosis is extensive. There is chronic inflammatory infiltration, whereas there is no neo-angiogenesis, lympho-vascular invasion, perineural invasion, and hemorrhagic area. There is a myometrial infiltration greater than 50%. a): It shows the histological tumor tissue (T) in contact with healthy histological tissue (H). b): histological tissue slice shows that adenocarcinoma (T) is characterized by the lack of an intermediate stroma and by cells arranged in a disordered way. c): histological tissue slice shows that healthy tissue (H) comprises neatly arranged cells.

**Table S1** Subjects specifications.

| **Subjects** | | **Number** | **Age [years]** | **BMI [Kg/m2]** | **Menopause** | **Hystology type** |
| --- | --- | --- | --- | --- | --- | --- |
| Healthy |  | 20 | 59 ± 7 | 25 ± 3 | All (=20) |  |
| EC |  | 18 | 72 ± 10 | 30 ± 5 | All (=18) |  |
|  | G1 | 3 |  |  |  | Endometroid |
|  | G2 | 6 |  |  |  | Endometroid |
|  | G3 | 5 |  |  |  | Endometroid |
|  | G3s | 4 |  |  |  | Serous papillary |

**Table S2** Average values of the parameters obtained from the Kurtosis model in the tumour zone (T), in the area immediately outside the tumour endometrium (PT), and the healthy endometrium (H).

| **ROI** | K ± $\sigma_{K}$ | D ± $\sigma_{D}$ ($\times{10}^{-3} \frac{{mm}^{2}}{s}$) |
| --- | --- | --- |
| H | 0.76 ± 0.08 | 1.09 ± 0.05 |
| T | 1.13 ± 0.08 | 0.87 ± 0.05 |
| PT | 1.64 ± 0.25 | 0.86 ± 0.08 |

**Table S3**: Differences and Cohen’s d values between the parameters divided by ROI (healthy H, tumor T, and peritumor PT).

|  | **K** | | **D** | |
| --- | --- | --- | --- | --- |
| **ROI** | **Difference** | **Cohen's d** | **Difference** | **Cohen's d** |
| H-T | -0.376 | 1.038 | 0.212 | 1.013 |
| H-PT | -0.887 | 1.162 | 0.226 | 0.776 |
| T-PT | -0.512 | 0.658 | 0.014 | 0.051 |

**Table S4**: Parameters of ROC curve analysis and classifier at Youden Index for each fold.

|  | **Accuracy** | **Precision** | **Recall** | **F1 score** |
| --- | --- | --- | --- | --- |
| H/T |  |  |  |  |
| fold 1 | 0.88 | 0.80 | 1 | 0.89 |
| fold 2 | 1 | 1 | 1 | 1 |
| fold 3 | 0.75 | 1 | 0.50 | 0.67 |
| fold 4 | 0.86 | 1 | 0.67 | 0.80 |
| fold 5 | 0.86 | 0.75 | 1 | 0.86 |
| H/PT |  |  |  |  |
| fold 1 | 1 | 1 | 1 | 1 |
| fold 2 | 1 | 1 | 1 | 1 |
| fold 3 | 0.88 | 0.80 | 1 | 0.89 |
| fold 4 | 1 | 1 | 1 | 1 |
| fold 5 | 1 | 1 | 1 | 1 |
